# 

**Published:** 1829-10

**Authors:** 


### MONTHLY LIST OF MEDICAL BOOKS.

[*Medical Works cannot be entered on this List except a copy be sent for the purpose; the titles of Books having frequently been transmitted to us, as published, which have not appeared for weeks, or even months, after.*]

Medical Botany, No. XXXIII. By John Stephenson, m.d. f.l.s. &c. and J. M. Churchill, f.l.s. &c.—Tilt, Fleet street.

In this Number there are well-executed engravings of the Pistacia Lentiscus, Origanum Vulgare, and Gentiana Lutea; together with a very complete account of their qualities and chemical properties, their medical properties and uses.

Atlas of Delineations of Cutaneous Eruptions; illustrative of the Descriptions in the Practical Synopsis of Cutaneous Diseases of Thomas Bateman, m.d. f.l.s. By A. T. Thomson, m.d. f.l.s. Professor of Materia Medica in the University of London, &c.—Longman, 1829.

In our review of Dr. Thomson's much-improved edition of Bateman's "Synopsis," we have expressed our opinion of the elegance, accuracy, and extreme utility of this "Atlas," in which various cutaneous diseases are illustrated for the first time. The plan of marking on each plate the commencement, progress, and termination of each eruption, renders the different delineations particularly useful to the student.

Pathological and Practical Researches on Diseases of the Brain and the Spinal Cord. By JOHN ABERCROMBIE, M.D. &c. Second Edition, enlarged. —8vo. pp. 476. Edinburgh and London, 1829.

The Mother's Monitor; or, Nursery Errors. By JAMES QUILTER RUMBALL, Surgeon, &c.—12mo. pp. 121. Wilson, London, 1829.

If the voice of this "Monitor" had never been heard, mothers would have had no cause for lamentation. Our readers will be satisfied with the following specimen of Mr. Rumball's correction of nursery errors: "Vomiting the food is a preventative of evil, requiring no remedy, and, *as it takes place when the stomach is too full, we need not be fearful of overloading it!!*" (Page 30.)

Elements of Practical Midwifery; or, Companion to the Lying-in Room. With Plates. By CHARLES WALLER, Consulting Surgeon to the London and Southwark Midwifery Institution, and Lecturer on Midwifery and the Diseases of Women and Children, at the Medical School, Aldersgate street.— 18mo. pp. 135. Highley, London, 1829.

### NOTICES.

*Communications have been received from* Dr. ARROWSMITH, Mr. JEWEL, Mr. COSTELLO, Dr. BLAKE, Dr. ROBERTS, &c.

*The Editors are obliged to* Dr. BLAKE *for his kind offer: they will at all times be happy to receive communications from him.*

*The Memorial, of which "the subject ought to rouse every medical man in England," appears to the Editors to be too entirely of a personal nature to produce the general excitement the memorialist expects.*

